# Foreign Body Aspiration in Children—Diagnostic Clues through a Clinical Case

**DOI:** 10.3390/pediatric14010012

**Published:** 2022-02-10

**Authors:** Elisabetta D’Addio, Pier Luigi Palma, Anna Di Sessa, Stefano Guarino, Pierluigi Marzuillo, Andrea Apicella

**Affiliations:** 1 Department of the Women, Children and General and Specialist Surgery, Università Degli Studi Della Campania “Luigi Vanvitelli”, 80138 Naples, Italy; elisabettadaddio3@gmail.com (E.D.); pieropalma2710@gmail.com (P.L.P.); anna.disessa@unicampania.it (A.D.S.); stefano.guarino@policliniconapoli.it (S.G.); 2 Peditric Emergency Department, A.O.R.N Santobono-Pausilipon Santobono, 80129 Naples, Italy; andrea.apicella@hotmail.com

**Keywords:** children, foreign body aspiration, clinical presentation

## Abstract

Foreign body aspiration is common in the pediatric age group, especially in males. Despite the high frequency of this potentially life-threatening event, it is not always easy to recognize it given the high variability of the clinical presentation and the potential of “pauci-symptomatic” inhalation. Moreover, a variable latency of the onset of symptoms since the moment of aspiration may be possible determining difficulties in the identification of the inhalation on an anamnestic basis. We describe the case of a 19-month-old boy who accessed the emergency room initially for a head trauma. The clinical evaluation, however, revealed an unexplained serious respiratory distress needing tracheal intubation. After our evaluation, we hypothesized that the severe respiratory distress determined an altered state of consciousness with following head trauma. The radiological findings raised the suspicion of foreign body aspiration for the presence of an atelectasis of the entire left lung. The computed tomography showed an abrupt interruption of the main bronchus at 12 mm from the hull. The following bronchoscopy identified an almond of 2 cm. We will review the literature to underline the diagnostic issues behind foreign body aspiration in children by highlighting the diagnostic clues that are helpful for emergency clinicians in the management of this condition.

## 1. Introduction

Foreign body aspiration (FBA) is a frequent problem in the pediatric age, being one of the most common causes of morbidity and mortality, especially in children under 3 years of age [1]. The clinical presentation of foreign body inhalation is often less specific but very insidious in some cases. We report the case of a 19-month-old boy who was diagnosed for FBA after accessing the emergency room initially for a head trauma. Moreover, we will review the literature to underline the diagnostic issues behind the FBA in children underlining the diagnostic clues able to help emergency clinicians in the management of this condition.

## 2. Case Presentation

**Figure 1:**
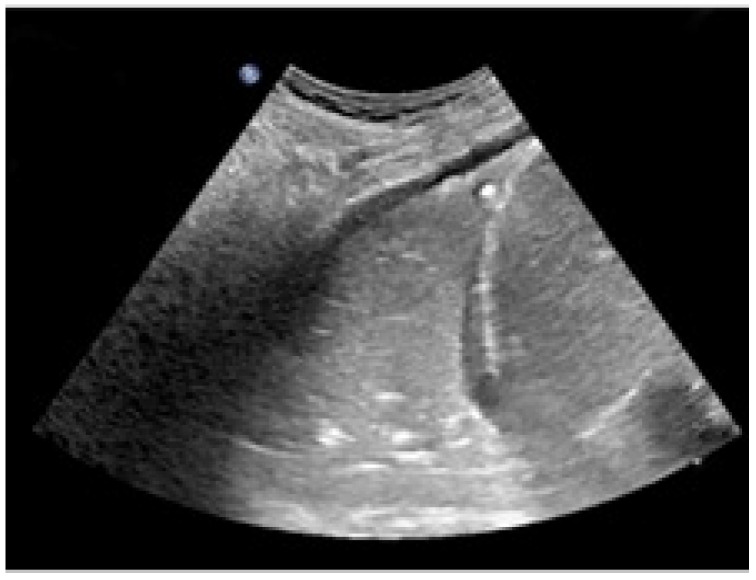
Left lung ultrasound showing a consolidation of the lung and the absence of the normal A lines.

**Figure 2:**
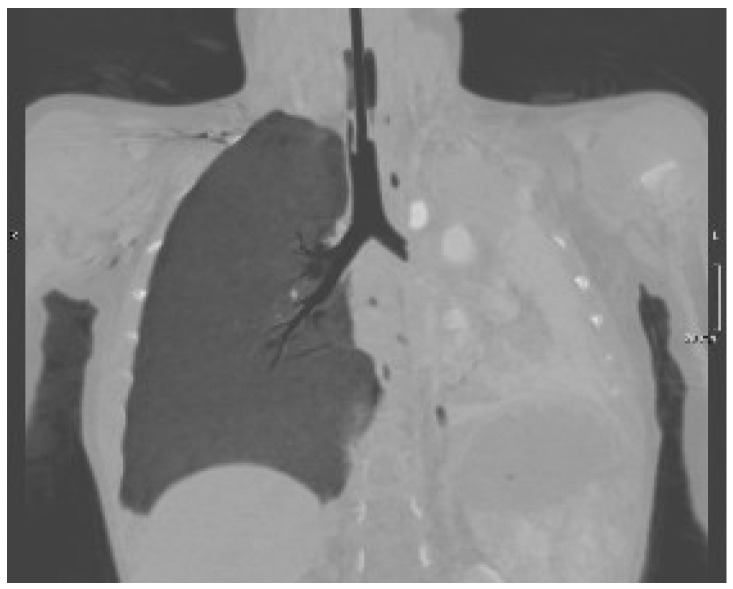
Chest CT scan showing a complete atelectasis of the left lung and an interruption of the main left bronchus.

A 19-month-old boy was admitted to the Emergency Department because he fell from his baby feeding highchair. This fall occurred in apparent well-being, without the presence of anticipatory signs or symptoms. First, he fell on his gluteus and then he banged his head (occiput) on the ground. He presented with vomiting (three episodes) and he was very irritable. His respiratory rate and heart rate were >60 breaths and >150 beats per minute, while oxygen saturation was <80%. Upon physical examination, the child was hydrated and conscious, but irritable. More importantly, we noted subcostal retractions, and, at the auscultation, decreased breath sounds in the left basal part of chest. The patient was ventilated with an AMBU balloon connected to an oxygen source and monitored with a pulse oximeter. Despite our intervention, oxygen saturation fell below 70% and the more we ventilated, the more the saturation dropped down. The lung ultrasound (Figure 1) showed the absence of the typical A lines and the consolidation of the lung, which was directly visualized as a solid parenchyma. On the basis of the poor clinical condition, the patient underwent orotracheal intubation with a cuffed endotracheal tube. After the baby was stabilized, he underwent a chest computed tomography (CT) showing complete atelectasis of the left lung with an interruption of the main left bronchus at 12 cm from bronchial bifurcation (Figure 2). An FBA was suspected as the mother also stated that the baby in the previous days had an intensive cough attack and disappeared within 24 h without any treatment. Therefore, a rigid bronchoscopy was performed and an almond of 2 cm in diameter in the main left bronchus was found and promptly removed.

The patient had never consumed almonds or other nuts before and in this circumstance ingested the almond by chance.

The baby had a progressive clinical improvement, and after 24 h, he was extubated and discharged from intensive care to be admitted to the general pediatrics ward for a few days with gradual and a total respiratory function recovery.

## 3. Discussion

FBA is a life-threatening condition for young patients. FBA is common especially between 1 and 3 years of age and our patient fell within this age range. Accordingly, Midulla et al. examined 70 patients undergoing bronchoscopy for FBA and found that 63 (90%) patients were less than 3 years old and 6 (8.6%) were even less than 1 year old. The majority of these patients (45/82; 54.9%) were of the male gender [1].

The higher incidence in the first years of life is probably related to the different behavior that children have in this period, as they are used to putting small objects into their mouths and they often cry and shout while parents try to feed them. Moreover, their molars are not yet developed, so they are not able to chew properly. Of note, an immature neuromuscular mechanism of airway protection represents a further contributing factor.

It is also widely documented that aspiration of a foreign body is a very dangerous condition for the life of young patients. With the improvement of diagnostic and treatment techniques, the mortality rate has decreased remarkably [2]. In fact, according to a French statistic in 1980, there were four cases of deaths from inhalation from an FBA per 10,000 inhabitants. In recent years, as a result of prevention campaigns and regulations concerning products designed for children, the infant mortality rate from FBA has decreased significantly, despite FBA remaining one of the main causes of infantile deaths [3]. In fact, according to the data provided by National Health and Family Planning Commission of the People’s Republic of China, suffocation following FBA is the ninth greatest cause of infantile death in China, with a death rate of 13.35/100,000 in 2012 [2].

Several different aspirated foreign bodies have been reported, both organic and inorganic. The most common aspirated foreign body is food, especially dried nuts or melon and sunflower seeds. The latter is more frequent in Middle Eastern countries, while nuts or inorganic bodies, such as plastic pieces of toys or coins, are mostly seen in Western countries. These differences are probably related to different cultural habits [3]. Other uncommon aspirated foreign bodies observed in the literature are vegetables, wooden or metal objects, beads, pins and small parts of school equipment such as pen caps [4]. Saki et al. studied 1015 patients who were diagnosed with FBA, and they found that the most common foreign body was dried fruit (63.87%), followed by other food-grade material (11.4%), peanuts (9.8%), bone (5.3%), metal objects (4.4%), plastic objects (2.4%) and other components in lower frequencies [4]. In our patient, the foreign body we found was an almond, which is very frequently found in the literature.

On the other hand, the localization in the left main bronchus as occurred in our case is less frequent compared to the localization in the right bronchus. Midulla et al., indeed, found that out of the 70 patients diagnosed with FBA, in 42 (60%), the foreign body was located in the right bronchial tree, in 23 (32.8%) the foreign body was located in the left bronchus, in three patients (4.3%) it involved the right and the left lung, and in two children (2.9%) the foreign body was located just below the vocal cords [1]. It could be supposed that the localization in the left main bronchus in our patient was a consequence of the push on the inhaled almond by the emergency ventilation. Commonly, most foreign bodies are found in the right main bronchus for anatomic reasons. Indeed, the right bronchus is larger than the left one, and most importantly, the degree between the right one and the trachea is about 20–25° compared with 40–50° of the left one [3]. This helps the foreign body to fall in the right bronchus.

When the foreign body has a larger diameter, it is more likely to obstruct the higher portions of the airways such as the larynx or trachea, and in this case, the symptoms appear immediately after inhalation with signs of suffocation. In these cases, it is necessary to intervene quickly with first aid maneuvers. If the foreign body has a smaller diameter, then it is more likely to wedge in the more sloping portions of the bronchial tree and the most common symptoms and signs are coughing, localized wheezing and decreased localized breath sounds [5]. Nevertheless, these symptoms are present in less than 40% of children, so the clinical findings achieve a low diagnostic sensitivity so we can never exclude FBA in asymptomatic patients [6]. Saki et al. showed that of the 1015 patients who underwent bronchoscopy for suspected FBA, 73.03% had cough, 13.18% cyanosis, 4.6% dyspnea, 2.2% wheezing, 1.73% unresolved lung infections, 3.2% choking, and 0.89% stridor as presenting symptoms, while 1.13% of cases were asymptomatic [4]. This is the reason why FBA could often be misdiagnosed. Karacok et al. showed that 32 out of 654 patients who underwent bronchoscopy with unclear respiratory clinical picture presented FBA and had a previous misdiagnosis of bronchitis, pneumonia, tuberculosis or croup [7].

In general, symptoms can occur within 8 h of inhalation, as well as several days later, in some cases even after 180 days [4]. We can justify this variability with the pathogenetic mechanism that underlies the FBA. In the case of small objects, the small diameter not determining a complete obstruction can determine a valve mechanism with less severe clinical pictures. On the other hand, in the case of inhalation of dried fruit, the inflammatory reaction with the formation of edema or the water imbibition of the dried fruit can determine an obstacle to the passage of air with sudden onset of symptoms [8]. As expected, a greater gap between the inhalation and the onset of symptoms might affect the medical history recall, leading to a diagnostic delay with consequent prognostic implications.

In our case, the mother initially brought the patient to the emergency room for a head injury, without dwelling on the obvious respiratory issues. Furthermore, she reported no potential previous inhalation episodes, and only after the almond was found, she began to remember what may have happened. Due to an in-depth medical history, she informed us that the baby was likely to have inhaled the almond seven days earlier when he accidentally took the almond. The patient, in fact, had never consumed almonds or other nuts before.

In this case, we faced a diagnostic challenge, as we were disoriented by the reported head trauma, and we found the presence of respiratory distress that could not be traced back to a definite cause. In the case of our baby, it is possible that we inadvertently excluded the left lung at the time of ventilation as we were unaware of the underlying cause. However, our management would not have contravened the American Heart Association’s Pediatric Advanced Life Support (PALS) guidelines, according to which in case of a history of certain inhalation and the child stops breathing, precise ventilation is required in order to engage the foreign object in the main bronchus and to be able to take advantage of the patency of the other bronchus for the cardiopulmonary resuscitation (CPR).

In our case, the radiological examination was very suggestive, but the bronchoscopy was decisive for the diagnosis by allowing the identification and removal of the almond.

Regarding the radiological presentation patterns occurring in case of FBA, there is current consensus on their variety, although some features have been encountered more frequently [9]. Of the 64 children undergoing chest X-ray, Midulla et al. found localized air trapping (43.6%), atelectasis (40%), mediastinal shift (25.5%), normal radiograph (20%), visible foreign body (9.1%), and pneumonia (5.5%) [1]. However, the use of chest X-ray is controversial because many children have normal chest radiography (up to 20%), while localized air trapping and atelectasis are the most frequent radiological findings (up to 50%) [1]. Pulmonary ultrasound is highly operator-dependent but has the advantage of being X-ray free. Its role in the FBA is limited but could be useful for atelectasis detection. In fact, in the case of our baby, the lack of typical A lines and the presence of a parenchymal echogenicity allowed us in a short time to suspect the presence of a pulmonary atelectasis that could have justified the picture of incipient respiratory insufficiency. Low-dose chest CT is an effective and consistent tool for diagnosing FBA in children, with a sensitivity of 100% and specificity of 98%, so it should be used to prevent unnecessary bronchoscopies [10]. However, the radiological features lack specificity, being fundamental for the diagnosis of rigid bronchoscopy as both its diagnostic and therapeutic role.

This case report allows us to underline that FBA often represents a major diagnostic challenge since its recognition is not always so immediate and can sometimes even be confused with other conditions, leading to a delay in diagnosis with potential unfavorable prognostic implications. For this reason, clinicians should always keep in mind FBA when facing unclear or persisting pulmonary symptoms [11].

## Data Availability

The study did not report any data.
